# Characteristics of *Sunsik*, a Cereal-Based Ready-to-Drink Korean Beverage, with Added Germinated Wheat and Herbal Plant Extract

**DOI:** 10.3390/foods9111654

**Published:** 2020-11-12

**Authors:** Bo Ram Kim, Seung Soo Park, Geum-Joung Youn, Yeon Ju Kwak, Mi Jeong Kim

**Affiliations:** 1 Interdisciplinary Program in Senior Human Ecology, Changwon National University, Changwon 51140, Korea; kinj56@daum.net (B.R.K.); pssll03@naver.com (S.S.P.); 2 Research Institute of GH Biofarm, Agricultural Corporation Gagopa Healing Food, 177, Samgye-ro, Naeseo-eup, Masanhoewon-gu, Changwon 51219, Korea; ygj@kfoofs.kr (G.-J.Y.); kyjred@kfoods.kr (Y.J.K.); 3 Department of Food and Nutrition, Changwon National University, Changwon 51140, Korea

**Keywords:** cereal-based ready-to-drink beverage, convenient meal replacement (CMR), germinated wheat, response surface methodology (RSM), gamma-amino butyric acid (GABA), antioxidant properties

## Abstract

The purpose of this study was to develop a formulation of *Sunsik* with improved health benefits by adding germinated wheat (GW) and herbal plant extract (HPE) using a response surface methodology (RSM). The central composite experimental design (CCD) was used to evaluate the effects of *Sunsik* with added HPE (2–4%) and GW (10–20%) on total phenolic content (TPC), total flavonoid content (TFC), Trolox equivalent antioxidant capacity (TEAC), 2,2-diphenyl-1-picrylhydrazyl (DPPH) radical scavenging capacity, gamma butyric acid (GABA) content, total color changes (△E), browning index (BI), water absorption index (WAI), and water solubility index (WSI). As a result of the CCD, the independent and dependent variables were fitted by the second-order polynomial equation, and the lack of fit for response surface models was not significant except in relation to WSI. The GABA content, TPC, and TEAC were more adequate for a linear model than for a quadratic model, and they might be affected by GW rather than HPE. Alternatively, the TFC, DPPH radical scavenging capacity, WAI, WSI, △E, and BI were fitted with quadratic models. The optimum formulation that could improve antioxidant and physicochemical properties was *Sunsik* with 3.5% and 20% added HPE and GW, respectively.

## 1. Introduction

Recently, the increase in single-person and double-income households has shifted consumers’ eating behaviors toward the increased consumption of home meal replacements (HMRs) or convenient meal replacement (CMRs) [1]. As ready-to-eat foods, CMRs are a more convenient and simpler meal replacement than HMRs, and they could reduce meal preparation and eating time. The CMR market quadrupled from $600 million in 2009 to $2.3 billion 2019. In Korea, the proportion of single-person households is expected to reach 35% of the total population in 2030, and the CMR market is expected to continue to grow. 

The types of CMR products are diversifying, such as to include liquid and powder grains, porridges, and cereal bars. Among them, cereal-based beverages are a representative CMR product consumed worldwide because they provide an efficient means to increase the intake of essential nutrients among busy modern people. A few studies investigated the physicochemical and health-conscious properties of various cereal beverages [2,3]. Bembem and Agrahar-Murugkar [2] reported that millet-based ready-to-drink beverages improved radical scavenging activity, total phenolic content (TPC), and viscosity in the geriatric population. In another study, multigrain beverages prepared with barley, oats, buckwheat, and red rice were identified as providing additional health benefits, such as phenolic content and soluble fiber, to consumers [3].

*Sunsik* has been consumed for a long time as a cereal-based ready-to-drink beverage in Korea. It is made of partially raw or thermal-processed and dried agricultural and marine products [4]. The most common ingredients of *Sunsik* are roasted brown rice, barley, adlay, oat, and black beans [5]. With the recent increase in the demand for healthy foods, much research has reported that additional ingredients, such as various dried vegetables, nuts, and fruits, could be added to *Sunsik* to offer more health-conscious nutrients [6,7,8]. For example, Park [8] reported that *Sunsik* with added mealworm was higher in antioxidant capacities and in consumer preference than a control *Sunsik*. Regarding the quality of ready-to-drink of *Sunsik*, it should disperse and dissolve well in water or milk within a few minutes. Koh, Jang, and Surh [6] reported that fermented *Sunsik* had a higher soluble solid content, oxidative stability, and amino acids than unfermented *Sunsik*, resulting in an improved solubility and nutrient content. Although several studies reported enhancements in the quality and nutrient content of *Sunsik*, there is limited information on the health benefits of *Sunsik* with added germinated wheat (GW) and herbal plant extract (HPE). 

Germination has been identified as an effective processing method to improve the nutritional quality and health-related compounds of cereal [9]. In numerous studies, gamma amino butyric acids (GABA) and phenolic acid compositions were increased as the germination time of wheat increased, suggesting the possibility of GW as a health-conscious ingredient [10,11,12]. In addition, Dhillon et al. [13] found that the antioxidant activity of and consumer preference for breads were improved when GW flour at 30 °C for 72 h was partially used to make bread. The changes in the physiological and biochemical properties of GW might be due to the activation of endogenous enzymes that break down starch and protein into small molecules [14,15]. The activation of endogenous enzymes may also play a role in increasing the solubility of *Sunsik* with added GW when it mixes with water or milk. In addition, plant herbal medicines, such as *Achyranthes aspera*, safflower seed, and Acanthopanax, have been used for the prevention of various diseases in traditional treatments in Asian countries [16,17]. It is known that safflower seeds are rich in lignin, flavonoid, and serotonin and have excellent effects on bone diseases, such as osteoporosis [18]. As previously published in many studies, the extracts of *A. aspera* and *Acanthopanax* showed a reduced inflammatory effect and antioxidant capacities [19,20,21,22]. The above-mentioned herbal plant medicines are used not only for therapeutic purposes, but also by adding them to various foods in the form of extracts to increase the health-related functions in the food matrix, such as noodles, drinks, and cookies [23,24,25,26]. The HPEs, including *A. aspera*, safflower seed, and *Acanthopanax*, used in this study confirmed previously the pharmacological effects on osteogenic differentiation in human mesenchymal stem cells [27]. The mixture extracts of herbal plants were freeze-dried and then were used in various food products of Gagopa Healing Food Co., Ltd. (Changwon, Korea).

Currently, *Sunsik* with added GW flour and HPE is not available in the marketplace yet. Thus, if GW and HPE are added to commercial *Sunsik*, which is conveniently used as ready to drink beverage, the new *Sunsik* product might be more beneficial to health. The purpose of this study was to determine the optimum formula amounts of GW flour and HPE powder for new *Sunsik* products as cereal-based ready-to-drink beverages. To determine the optimum formulation of *Sunsik*, the response surface methodology (RSM) was adopted using a central composite experimental design (CCD). The antioxidant capacities, GABA, water absorption index (WAI), water solubility index (WSI), total color changes (ΔΕ), and browning index (BI) were analyzed to optimize the health-conscious nutrients and quality of *Sunsik*; then, the newly optimized *Sunsik* was compared with control *Sunsik* in terms of various health-conscious and physicochemical properties.

## 2. Materials and Methods 

### 2.1. Materials

The *Sunsik* and HPE were provided from Gagopa Healing Food Co., Ltd. (Changwon, Korea). The main ingredients of *Sunsik* consisted of 30% barley, 30% brown rice, 20% adlay, 10% black bean, and 10% oat. In general, each cereal was steamed and then dry-roasted. The four roasted cereals were pulverized in a batch for a production of the Sunsik. The Sunsik used in this study is being sold on the market. Gagopa Healing Food Co., Ltd. (Changwon, Korea) found effects of HPE on osteogenic differentiation through preliminary studies, and the results already published [27]. The HPE used in this study is composed of safflower seed (85%), *A. aspera* (5%), manyprickle acanthopanax (5%), and *Kalopanax septemlobus* (5%) [27]. In addition, the GW used in this study was prepared according to preliminary experiments. Anzunbaengi wheat, which was cultivated in Jinju, Korea, was germinated at 17.6 °C for 46.18 h to enhance GABA. After germination, the GW was freeze-dried and then grounded to powder. To develop a cereal-based ready-to-eat beverage to enhance health-related properties, *Sunsik* was formulated with HPE and GW to maximize GABA and antioxidant capacities. The ranges of HPE and GW used in this study were 2–4% and 10–20%, respectively, and the ranges were determined based on samples of five points or more as a result of consumer acceptability (nine-point hedonic scale) of *Sunsik* with added HPE or GW, respectively.

### 2.2. Experimental Design and Optimization of the Formulation 

The amounts of HPE and GW were optimized using a CCD of an RSM [28]. The independent values were studied at five different levels (− α, −1, 0, + 1, and + α), and the actual levels are presented in Table 1.

Table 1 and they were evaluated to maximize the GABA, total flavonoid content (TFC), TPC, 2,2-diphenyl-1-picrylhydrazyl (DPPH) radical scavenging capacity, Trolox equivalent antioxidant capacity (TEAC), and WSI and to minimize the WAI, ΔΕ, and BI. The effects of the two independent variables on the responses (Y) were modeled using the response surface regression, and they were predicted by the following Equation (1) [28]:

(1) $$Y_{k}=\beta_{0}+\beta_{1}X_{1}+\beta_{2}X_{2}+\beta_{12}X_{1}{\;X}_{2}+\beta_{11}X_{1}^{2}+\beta_{22}X_{2}^{2}$$

where β_0_ is a constant, β_1_ and β_2_ are the linear coefficients, β_12_ is the interaction coefficient, and β_11_ and β_22_ are the quadratic coefficients. X_1_ and X_2_ are the levels of HPE and GW, respectively. Y_k_ is the response variable, and each response variable is as follows; Y_1_ = GABA (µg/g), Y_2_ = TFC (µg CE/g), Y_3_ = TPC (µg GE/100g), Y_4_ = DPPH (µM TE/100g), Y_5_ = TEAC (mM TE/100g), Y_6_ = WAI, Y_7_ = WSI, and Y_8_ = ΔΕ, Y_9_ = BI. To validate the linear or quadratic model, each experimental data of independent variables was compared with the predicted values using the model developed in this study.

### 2.3. Extraction Procedure of Sunsik Samples

In total, 5 g of each *Sunsik* sample was extracted with 80% ethanol at 65 °C for 2 h, and the supernatants obtained by centrifugation (5000 rpm for 30 min) were evaporated to dryness at 45 °C using a nitrogen evaporator (Eyela MG-2200, Tokyo Rikakikai Co. Ltd., Tokyo, Japan). The dried extract was then re-dissolved with 80% ethanol into a final volume of 5 mL. The extract was used to determine the GABA, TEAC, DPPH, TFC, and TPC.

### 2.4. Gamma-Amino Butyric Acid (GABA)

The GABA contents of the *Sunsik* samples were determined according to the method described in Sharma et al. [29]. In brief, 0.1 mL of each extract was mixed with 0.2 mL of 0.2 M borate buffer and 1 mL of 6% phenol reagent. Then, 0.4 mL of 7.5% sodium hypochlorite was added, and the mixture was boiled for 10 min in a water bath. The samples were immediately cooled for 5 min, and the absorbance was measured using a spectrophotometer (EMC-11D-V Spectrophotometer, EMCLAB Instruments, Duisburg, Germany) at 630 nm. The GABA was used as a standard curve and prepared with a range of concentrations from 0 to 50 mg. Results were expressed as mg/g.

### 2.5. Total Flavonoid Content (TFC)

TFC was determined using the methods previously described by Dahl [30]. The extract of samples (250 µL) was added to 1.25 mL distilled water, and 70 µL of 5% sodium nitrite was added to the mixture. After 6 min, 150 µL of 10% aluminum chloride was added to the mixture. After 5 min, 0.5 mL of 1 N sodium hydroxide was added to the mixture. The absorbance was measured immediately at 510 nm. Distilled water was used as a blank. Catechin was used as a standard curve and prepared with a range of concentrations from 0 to 2.5 mg. The results were reported as catechin equivalents (CE) µg/g.

### 2.6. Total Phenolic Content (TPC) 

TPC was determined by the method described by de la Rosa et al. [31] with modifications. TPC was measured using the Folin-Ciocalteu method. In total, 100 µL of each extract was added to 2.5 mL of 10% Folin-Ciocalteu reagent, and the mixture was allowed to stand for 2 min. Then, 2 mL of 6% sodium carbonate was added to the mixture, and it was incubated at 50 °C for 15 min in a water bath. The absorbance was measured at 760 nm, and distilled water was used as a blank. Gallic acid was used as a standard curve and prepared with a range of concentrations from 0 to 50 mg. Results were expressed as gallic acid equivalents (GAE) mg/g.

### 2.7. DPPH Radical Scavenging Capacity 

The determination of the effect scavenging of the DPPH radical was based on a procedure previously described by Wong et al. [32]. A 0.1 mM DPPH solution diluted with 100% methanol was prepared. In addition, 0.1 mL of the sample and 1.9 mL of 0.1 mM DPPH were mixed well. The DPPH solution was allowed to stand for 30 min at room temperature in the dark. Then, the absorbance was measured at 515 nm, and 100% methanol was used as a blank. Furthermore, 10 mM Trolox was used as a standard curve and prepared with a range of concentrations from 0 to 500 μM. Results were expressed as μmol of Trolox equivalents (TE) μmol/100 g.

### 2.8. Trolox Equivalent Antioxidant Capacity (TEAC)

TEAC was performed as described by Simsek and El [33], with modifications. Briefly, an ABTS^+^ stock solution was prepared with 7.4 mM ABTS and 2.6 mM potassium persulfate and mixed. After, the mixture was allowed to stand for 16 h at room temperature in the dark. The ABTS^+^ stock solution was diluted with 100% methanol to an absorbance wavelength of 0.7 at 734 nm. Then, 2960 µL of the ABTS^+^ stock solution was added to 20 µL of the sample, and absorbance was measured after 7 min. Trolox was used as a standard curve and prepared with a range of concentrations from 0 to 1000 µg. Results were expressed as mmol of TE mmol/100 g.

### 2.9. Water Absorption Index (WAI) and Water Solubility Index (WSI)

The WAI and WSI of the optimized *Sunsik* and control samples were determined using methods previously described by Du et al. [34] with slight modifications. In total, 2.5 g of the sample was added to 30 mL of distilled water and mixed in a shaking water bath at 30 °C for 30 min. Then, the mixture was centrifuged at 3000 rpm for 15 min. The supernatant and remaining sediment from the mixture were weighted. The supernatant was decanted into an aluminum dish and dried at 105℃ overnight using a dry oven. The WAI and WSI were calculated as in the following equations, respectively.

(2) $$\mathrm{WAI}=\frac{\mathrm{weight}\mathrm{of}\mathrm{the}\mathrm{sediment}\left(g\right)}{\mathrm{weight}\mathrm{of}\mathrm{the}\mathrm{sample}\left(g\right)}$$

(3) $$\mathrm{WSI}\left(\%\right)=\frac{weight\;of\;dry\;solids\;from\;the\;supernatant\;\left(g\right)}{weight\;of\;the\;sample\;\left(g\right)}\times 100$$

### 2.10. Color Properties

The color values of the optimized Sunsik and control samples were determined with a CIE Lab system using a color meter (CR-400, Konica minolta sensing Inc., Osaka, Japan). It was calibrated with a white ceramic plate before measuring the sample. The total color changes (ΔΕ) and browning index (BI) were calculated as follows [35,36]:

(4) $$\Delta E=\sqrt{{\left(L_{0}^{*}-L^{*}\right)}^{2}+{\left(a_{0}^{*}-a^{*}\right)}^{2}+{\left(b_{0}^{*}-b^{*}\right)}^{2}}$$

(5) BI=[100(X−0.31)]/0.172

(6) $$X=\left(a^{*}+1.75L^{*}\right)/\left(5.645L^{*}+a^{*}-3.012b^{*}\right)$$

where $L_{0}^{*}$, $a_{0}^{*}$, and $b_{0}^{*}$ are color parameters for the control and $L^{*}$, $a^{*}$, and $b^{*}$ are color parameters for each Sunsik sample.

### 2.11. Apparent viscosity of Sunsik Samples

The apparent viscosity of the optimized *Sunsik* and control samples was measured using a digital rotary viscometer (WVS-0.1M, DAIHAN Scientific, Gang-Won-Do, Korea). First, 45 g of the sample was placed in a 500-mL beaker, and 300 mL of water or milk was poured in, followed by thorough mixing with a magnetic stirrer (MS-20D, DAIHAN Scientific, Gang-Won-Do, Korea). Finally, the thoroughly mixed sample was poured into a 250-mL beaker (SDS 2400, DONG SUNG science, Gang-Won-Do, Korea) and the viscosity of the sample was measured. When measuring the viscosity, the standard was measured when the torque value was close to 50%.

### 2.12. Cell Proliferative Effects of Sunsik Samples on Caco-2 and HepG2 Cells 

In total, 15 g of the *Sunsik* samples was extracted with 80% ethanol, evaporated to dryness at 45 °C, and re-dissolved in dimethyl sulfoxide (DMSO) according to a previously described method [37]. The Caco-2 (ATCC^®^HTB-37^TM^, Manassas, USA) cell was cultured in MEM (Hyclone Laboratories Inc., South Logan, UT, USA) with 10% or 20% fetal bovine serum (FBS, Welgene, Daegu, Korea) at 37 °C in a humidified incubator with 5% CO_2_. The cell proliferation of *Sunsik* extracts was determined by MTT (3-(4,5-dimethylthiazol-2-yl)-2,5-diphenyl tetrazolium bromide) assay. The cells (1 × 10_4_/well) were seeded in 96-well plates and then allowed to attach overnight. After overnight, the media included with *Sunsik* extracts were exchanged and incubated for 72 h. After 72 h of incubation, cell proliferation was determined using the MTT Cell Proliferation Assay kit (Roche Ltd., Mannheim, Germany) at 570–655 nm with a SpectraMax^®^i3 plate reader (Molecular Devices, Sunnyvale, CA, USA).

### 2.13. Data Analysis

The Design Expert software (version 11, State-Ease Inc., Minneapolis, USA) was used to analyze the experimental data for best fit model equations and to obtain response plots for each response variable. The combination of independent variables generating the highest overall desirability was selected as the optimum formulation. To validate the optimization process, the Sunsik was prepared using the optimum levels of independent variables and analyzed for the selected responses. The absolute residual error (%) was calculated using the experimental and predicted data through the following Equation (7):

(7) $$Absolute\;residual\;error\left(\%\right)=\frac{Actual\;value-Predicted\;value}{Actual\;value}\times 100$$

All experiments were carried out in triplicate, and ANOVA was performed to determine differences among the samples using the XLSTAT software (Addinsoft, Paris, France). When a difference among the samples was identified, the Student Newan–Keul’s (SNK) multiple comparison was performed to separate the means.

## 3. Results and Discussion

### 3.1. Fitting the Model and Statistical Analysis

The RSM is often used to determine the formulation ratio of a new product in the food industry. In this study, a CCD was applied to determine the optimum formulation of HPE and GW to prepare healthy Sunsik, a cereal-based ready-to-drink Korean beverage. The independent and dependent variables were fitted by linear or quadratic equations, and Table 2 shows the statistical results of the regression coefficients, R^2^, adjusted R^2^, lack of fit, and p values of the fitted models on analyzed responses by CCD. As shown in Table 2, the lack of fit for response surface models was not significant without the WSI, implying that the response surface models were adequately explained for predicting the relevant responses [28].

Among the responses, GABA, TPC, and TEAC were more adequate for a linear model than for a quadratic model. Because the β_2_ values of GABA (*p* < 0.01), TPC (*p* < 0.05), and TEAC (*p* < 0.01) differed significantly, the GABA, TPC, and TEAC contents of newly developed Sunsik might be affected by GW rather than HPE. The final equations of GABA, TPC, and TEAC as follows:

(8) GABA=2.09+0.017×HPE+0.1031×GW

(9) TPC=70.57−0.3237×HPE+2.21×GW

(10) TEAC=120.16+1.34×HPE+3.39×GW

As described in Table 2, the TFC, DPPH, WAI, WSI, ΔΕ, and BI were fitted with quadratic models. The final equations of TFC, DPPH, WAI, WSI, ΔΕ, and BI were coded as follows:

(11) $$\mathrm{TFC}=30.99+1.11\times \mathrm{HPE}+3.03\times \mathrm{GW}+1.18\times \mathrm{HPE}\times \mathrm{GW}-1.45\times {\mathrm{HPE}}^{2}-1.47\times {\mathrm{GW}}^{2}$$

(12) $$\mathrm{DPPH}=106.59+3.71\times \mathrm{HPE}+3.32\times \mathrm{GW}+2.39\times \mathrm{HPE}\times \mathrm{GW}-3.49\times {\mathrm{HPE}}^{2}-2.80\times {\mathrm{GW}}^{2}$$

(13) $$\mathrm{WAI}=1.85+0.0068\times \mathrm{HPE}-0.0196\times \mathrm{GW}-0.0394\times \mathrm{HPE}\times \mathrm{GW}+0.014\times {\mathrm{HPE}}^{2}+0.0263\times {\mathrm{GW}}^{2}$$

(14) $$\mathrm{WSI}=48.44-0.3332\times \mathrm{HPE}+4.52\times \mathrm{GW}+0.0882\times \mathrm{HPE}\times \mathrm{GW}-4.61\times {\mathrm{HPE}}^{2}-2.03\times {\mathrm{GW}}^{2}$$

(15) $$\Delta E=0.2224-0.2736\times \mathrm{HPE}+0.1071\times \mathrm{GW}-0.0362\times \mathrm{HPE}\times \mathrm{GW}+0.2708\times {\mathrm{HPE}}^{2}+0.1384\times {\mathrm{GW}}^{2}$$

(16) $$\mathrm{BI}=20.02+0.259\times \mathrm{HPE}+0.015\times \mathrm{GW}-0.1831\times \mathrm{HPE}\times \mathrm{GW}-0.0531\times {\mathrm{HPE}}^{2}+0.0061\times {\mathrm{GW}}^{2}$$

The higher values of R^2^ and adjusted R^2^ mean desirability of the model to explain the relationships between variables [28]. In this study, the responses with R^2^ values of 0.8 or higher were TFC, DPPH, WAI, and ΔΕ, indicating that the fitted equations adequately describe the effects of adding GW and HPE to Sunsik on each dependent variable.

### 3.2. Effects of Independent Values on Health-Conscious Properties 

**Figure 1:**
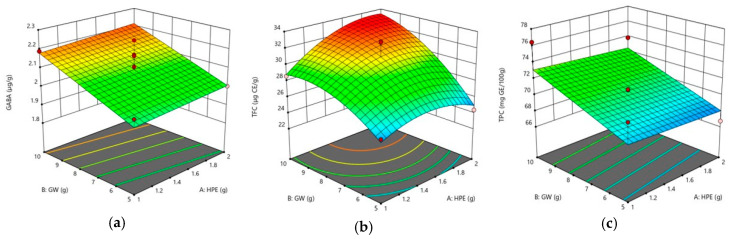
Three-dimensional response surface plots of the GABA content (**a**), TFC (**b**), and TPC (**c**). (GW: germinated wheat; HPE: herbal plant extract; GABA: gamma aminobutyric acid; TFC: total flavonoid content; TPC: total phenolic acid).

The GABA, TFC, and TPC contents and antioxidant capacities (DPPH radical scavenging capacity and TEAC) of differently formulated Sunsik samples by CCD are shown in Table 3. Significant differences among the 13 samples were found in the GABA (*p* < 0.01), TFC (*p* < 0.001), TPC (*p* < 0.001), DPPH (*p* < 0.05), and TEAC (*p* < 0.05) contents. The GABA content, TFC, and TPC are some of the major compounds that contribute to the antioxidant capacities, such as DPPH and TEAC [11,30,38]. The GABA content and TPC were in the ranges of 1.81–2.25 μg/g and 67–76 μg GE/100g, respectively. As shown in Table 2, the GABA content and TPC were significant in the β_2_ value (*p* < 0.01 for GABA and *p* < 0.05 for TPC) but not significant in the β_1_ value, indicating that the GABA content and TPC of Sunsik with added HPE and GW were influenced by increased GW. These results were also confirmed in the three-dimensional response surface plots of Figure 1a,c.

Conversely, the addition of HPE and GW had significant quadratic effects (*p* < 0.05 for β_11_ and *p* < 0.05 for β_22_) on TFC (Table 2). Figure 1b shows the three-dimensional response surface plots of TFC, implying the TFC of *Sunsik* is increased by both HPE and GW.

**Figure 2:**
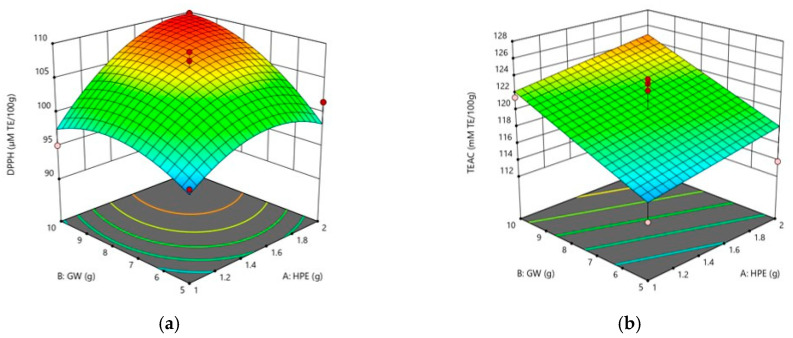
Three-dimensional response surface plots of DPPH (**a**) and TEAC (**b**).

The antioxidant properties of 13 Sunsik samples corresponding to the experiments generated by the CCD were determined by DPPH and TEAC (Table 3). The DPPH and TEAC values of the samples differed significantly (both *p* < 0.05) and were in the ranges of 96–110 µM TE/100g and 113–127 mM TE/100 g, respectively. As presented in Table 2, the DPPH value was fitted with a quadratic model while TEAC value was fitted with a linear model. The comprehensive effects of the dependent variables (HPE and GW) on the antioxidant properties of Sunsik are represented by the response surface plots in Figure 2.

The Sunsik samples with higher antioxidant activities contained relatively high GABA content, TPC, and TFC. These results are in agreement with previous studies [11], which reported a higher antioxidant capacity of the samples containing higher GABA content, TPC, and TFC. The increments of TPC and GABA content in Sunsik samples could be explained by the addition of GW. Chen et al. [39] reported that phenolic contents in GW increased by lignin synthesis during germination. In addition, another study explained that the GABA content in GW increased via the decarboxylation of L-glutamate [11]. Safflower seed, a major material of HPE, has protective effects against osteoporosis and a beneficial effect on atherogenic risk through various phenolic compounds, such as lignin and flavonoids [25]. Recently, the antioxidant, anti-cancer, anti-inflammatory effects of safflower seeds have been identified by a few studies [25,40,41]. 

### 3.3. Effects of Independent Values on Physicochemical Properties

**Figure 3:**
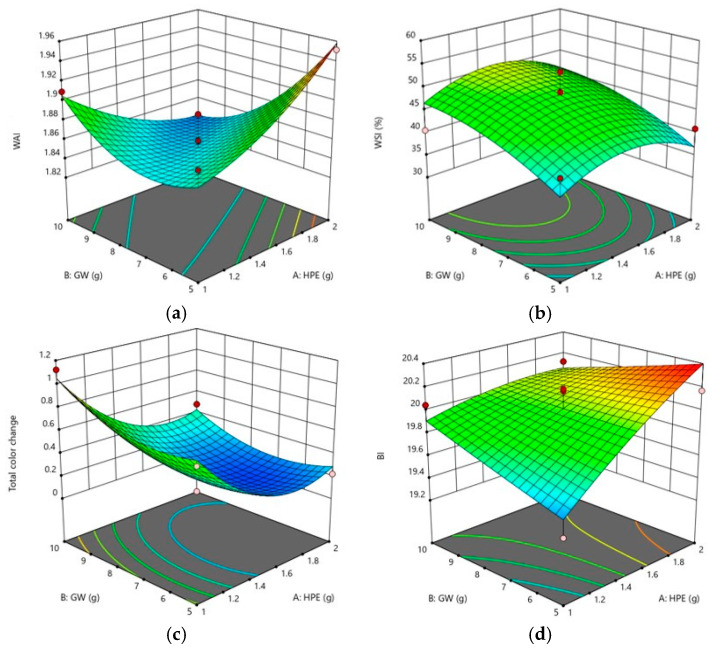
Three-dimensional response surface plots of the WAI (**a**), WSI (**b**), ΔΕ (**c**), and BI (**d**).

The WAI and WSI are important parameters in powdered cereal-based beverages, such as Sunsik, which is eaten by dissolving in milk or water. The WAI and WSI values of the Sunsik samples tested in this study are presented in Table 4. The WAI values of the Sunsik samples were in the range of 1.82–1.95 and did not differ significantly (Table 4). Although there was no statistically significant difference in the WAI values of Sunsik samples, they tended to increase as the amount of HPE increased (Figure 3a). The WAI value of reconstituted powder, such as Sunsik examined in this study, might play a role in preventing its dissolution in milk or water [42]. As shown in the WAI results of Table 2, the linear coefficients of HPE (β_1_) and GW (β_2_) were 0.0018 and −0.0195, respectively, implying that GW in newly formulated Sunsik had a negative effect. The WSI is the amount of soluble components released from the Sunsik samples, and the values ranged from 32% to 59% (Table 4). The WSI values of Sunsik with 1.5 g of added HPE and 11.04 g of added GW were the highest among the samples, suggesting the contribution of GW to the solubility of the newly formulated Sunsik samples (Figure 3b).

Significant differences were observed in the ΔΕ (*p* < 0.001) and BI (*p* < 0.01) values among the newly formulated Sunsik samples (Table 4), which were in the ranges of 0.22–1.13 and 19.2–20.3, respectively. In the results of the regression coefficients, the HPE addition negatively affected and the GW addition positively affected the ΔΕ of the newly formulated Sunsik. The three-dimensional response surface plots also showed a similar trend (Figure 3c), indicating that the color of the newly formulated Sunsik was mostly affected by a higher GW amount than HPE amount. Such a result was expected, as more GW (10–20%) was added to Sunsik than HPE (2–4%). The color affects consumer perceptions of various foods or beverages, and color changes or a brown color during processing or cooking might negatively affect consumer preferences [43]. As shown in Figure 3d, the brown color changes of Sunsik were the result of adding HPE. In a preliminary experiment to determine the range of the HPE amount, consumers tended not to prefer Sunsik with more than 4% HPE added due to its darkened color.

### 3.4. Optimization and Validation 

Cereal-based products like Sunsik are often developed with the addition of two or more ingredients to provide additional health benefits to consumers. In this study, both GW and HPE had a significant effect on the health-related properties and physicochemical characteristics of Sunsik. The additions of GW and HPE in newly formulated Sunsik were response specific. Thus, optimization is needed to attain a formulation with the desired characteristics concerning all the responses. 

*Sunsik*, a cereal-based ready-to-drink beverage, was optimized considering maximized properties, such as GABA, TFC, TPC, DPPH, TEAC, and WSI. By contrast, WAI, ΔE, and BI were minimized in Sunsik products. The optimized formula of Sunsik developed in this study was 10 g of GW, 1.79 g of HPE, and 38.21 g of Sunsik corresponding to the highest desirability of 0.719. In addition, the predicted and actual values for optimized formulations of Sunsik are presented in Table 5. Both the predicted and actual values were compared and were verified using absolute residual error values (Table 5). The errors for the responses were found to be less than 5% without ΔE. This indicated the precision of the developed and optimized regression models for the newly formulated Sunsik products.

### 3.5. Health-Conscious and Physicochemical Properties of Optimized Sunsik

Because the purpose of this study was to develop a newly formulated Sunsik containing GW and HPE to provide health benefits over the commercially available Sunsik, various properties of commercial and optimized Sunsik were compared. The health-conscious and physicochemical properties of both Sunsik samples are presented in Table 6. The GABA content, TPC, and TFC might be major constituents contributing to the antioxidant capacities and antiproliferative cancer cells [38]. Significant differences between the commercial and optimized Sunsik samples with respect to the GABA content (*p* < 0.001), TFC (*p* < 0.001), and TPC (*p* < 0.001) were observed (Table 6). The optimized Sunsik contained more GABA (2.23 μg/g) content, TFC (33.75 μg CE/ 100g), and TPC (73.75 μg GE/100g) than commercial Sunsik (GABA: 1.7 μg/g; TFC 19.8 μg CE/100 g; TPC: 54.4 μg GE/100g), confirming health benefits of optimized Sunsik compared to commercial Sunsik. 

In addition, the DPPH (*p* < 0.001) and TEAC (*p* < 0.001) of optimized Sunsik, to which 10 g of GW and 1.79 g of HPE were added, increased significantly compared to commercial Sunsik. Numerous studies have been developed new product with more antioxidant or antiproliferative activities to contribute health benefits of consumed products [7,8,38]. According to Kim and Kim [38], cereal products containing higher phenolic or flavonoid contents had higher antioxidant capacities. In this study, optimized Sunsik contained higher TPC, TFC, DPPH, and TEAC values than the commercial Sunsik. Similar trends were observed in terms of the proliferative activities of cancer cells. The relative proliferative effects on Caco-2 and HepG2 cells after treatment with an extract of the samples are shown as the median effective dose (EC_50_) in Table 6. The EC_50_ values of optimized Sunsik for Caco-2 and HepG2 cells were 45.7 and 35.2 mg/mL, respectively. Commercial Sunsik was relatively high in EC_50_ values of Caco-2 (97.9 mg/mL) and HepG2 (76.2 mg/mL) cells compared to those of optimized Sunsik (Caco-2: 45.7 mg/mL; HepG2: 35.2 mg/mL), indicating relatively low antiproliferative activities. Many studies have reported that foods or beverages with antioxidant activities have cancer-protective effects [37], suggesting that cereal-based beverages could inhibit cancer cell growth. In this study, optimized Sunsik added with GW and HPE showed higher antioxidant capacity and antiproliferative activity than commercial Sunsik.

The WAI, WSI and viscosity of optimized Sunsik with added GW and HPE were compared to commercial Sunsik, and the results are shown in Table 6. The WAI and viscosity of cereal-based beverages are important quality factors [3,4]. According to the finding of Fernandes, Sonawane, and Arya [3], the high absorbing properties in cereal-based beverages resulted in increased viscosity, and high viscosity negatively affected mouthfeel and overall acceptability in sensory tests. According to the results of the current study, the WAI and viscosity of optimized Sunsik with added GW and HPE were less than that of the commercial Sunsik sample. The low WAI and viscosity might contribute to the solubility of Sunsik, which is eaten by dissolving in milk or water, showing higher WSI values in optimized Sunsik than in commercial Sunsik. 

## 4. Conclusions

This study showed that the CCD and RSM could be used to optimize the formulation of *Sunsik*, a cereal-based ready-to-eat beverage. RSM predicted that a *Sunsik* formula of 10 g GW, 1.79 g HPE, and 38.21 g *Sunsik* would provide a better quality with more health-conscious and physicochemical characteristics. The optimized *Sunsik* is characterized by higher GABA, TPC, TFC, DPPH, TEAC, and WAI values than commercial *Sunsik*. The EC_50_ of cancer cells, WAI, and viscosity were low in optimized *Sunsik* compared to commercial *Sunsik*. Overall, *Sunsik* with 10 g of added GW and 1.79 g of added HPE might increase various health-related components and biological activities while maintaining the quality of the cereal-based beverage.

## Figures and Tables

**Table 1:** The coded levels and actual values of 13 experiments formulated with a central composite design (CCD).

Experiment No.	Coded Levels		Actual Values		
	X_1_ (HPE, g)	X_2_ (GW, g)	X_1_ (HPE, g)	X_2_ (GW, g)	*Sunsik* (g)
1	−1	−1	1	5	44
2	1	−1	2	5	43
3	−1	1	1	10	39
4	1	1	2	10	38
5	α(−)	0	0.79	7.5	41.71
6	α(+)	0	2.21	7.5	40.29
7	0	α(−)	1.5	3.96	44.54
8	0	α(+)	1.5	11.04	37.46
9	0	0	1.5	7.5	41
10	0	0	1.5	7.5	41
11	0	0	1.5	7.5	41
12	0	0	1.5	7.5	41
13	0	0	1.5	7.5	41

**Table 2:** The regression coefficients, R square, adjusted R square, lack of fit, and *p* values of the fitted models on dependent variables.

		Health Conscious Properties					Physicochemical Properties			
		GABA	TFC	TPC	DPPH	TEAC	WAI	WSI	ΔΕ	BI
Constant	β_0_	2.09	30.99	70.57	106.59	120.16	1.85	48.44	0.2224	20.02
Linear	β_1_	0.0170	1.11	−0.32	3.71 **	1.34	0.0068	−0.3332 *	−0.2736 **	0.2590 **
	β_2_	0.1031 **	3.03 ***	2.21 *	3.32 **	3.39 **	−0.0196 *	4.52	0.1071 *	0.0150
Quadratic	β_11_		1.18 *		2.39 **		−0.0394	0.0882 *	−0.0362 ***	−0.1831
	β_22_		−1.45 *		−3.46 **		0.0140 *	−4.61	0.2708 **	−0.0531
Interaction	β_12_		−1.47		−2.80		0.0263 **	−2.03	0.1384	0.0061
R^2^		0.546	0.889	0.487	0.886	0.563	0.828	0.583	0.952	0.702
Adjusted R^2^		0.455	0.809	0.384	0.804	0.476	0.805	0.285	0.917	0.489
Lack of Fit (*p* value)		0.196	0.745	0.052	0.094	0.228	0.533	0.041	0.452	0.193
*p* value		0.019	0.003	0.004	0.003	0.015	0.013	0.203	0.0002	0.075

*^,^ **^,^ *** significantly differ at *p* > 0.05, *p* < 0.01, and *p* < 0.001, respectively. β_1_: herbal plant extract; β_2_: germinated wheat.

**Table 3:** The experimental values of the health-conscious variables for each independent variable.

Experiment No.				GABA ** (Y_1_, µg/g)	TFC *** (Y_2_, µg CE/g)	TPC *** (Y_3_, µg GE/100 g)	DPPH * (Y_4_, µM TE/100 g)	TEAC * (Y_5_, mM TE/100 g)
	HPE (g)	GW (g)	*Sunsik* (g)					
1	1	5	44	2.01 ± 0.09 ^ab^	26 ± 2.30 ^bc^	71 ± 2.23 ^cd^	96 ± 3.7 ^b^	113 ± 3.93 ^b^
2	2	5	43	2.00 ± 0.08 ^ab^	24 ± 1.21 ^c^	67 ± 0.44 ^f^	102 ± 2.9 ^ab^	114 ± 3.70 ^b^
3	1	10	39	2.19 ± 0.13 ^a^	29 ± 3.68 ^abc^	76 ± 0.88 ^a^	95 ± 3.1 ^ab^	122 ± 5.48 ^ab^
4	2	10	38	2.14 ± 0.16 ^a^	32 ± 3.34 ^ab^	74 ± 0.97 ^b^	110 ± 9.2 ^ab^	122 ± 3.30 ^ab^
5	0.79	7.5	41.71	1.98 ± 0.06 ^ab^	26 ± 0.92 ^abc^	69 ± 0.70 ^de^	96 ± 6.9 ^b^	116 ± 4.27 ^b^
6	2.21	7.5	40.29	2.12 ± 0.04 ^a^	31 ± 2.80 ^abc^	72 ± 0.09 ^c^	103 ± 9.4 ^b^	123 ± 2.86 ^ab^
7	1.5	3.96	44.54	1.81 ± 0.15 ^b^	22 ± 2.00 ^c^	68 ± 0.40 ^ef^	94 ± 6.9 ^ab^	119 ± 3.60 ^ab^
8	1.5	11.04	37.46	2.17 ± 0.08 ^a^	33 ± 3.48 ^abc^	72 ± 0.88 ^c^	107 ± 8.3 ^a^	127 ± 5.22 ^a^
9	1.5	7.5	41	2.09 ± 0.04 ^a^	30 ± 2.54 ^ab^	69 ± 0.99 ^de^	108 ± 8.8 ^ab^	120 ± 2.36 ^ab^
10	1.5	7.5	41	2.11 ± 0.11 ^a^	30 ± 1.43 ^ab^	68 ± 0.02 ^ef^	106 ± 1.8 ^ab^	123 ± 4.42 ^ab^
11	1.5	7.5	41	2.17 ± 0.11 ^a^	30 ± 1.90 ^abc^	69 ± 0.71 ^de^	105 ± 2.5 ^ab^	124 ± 4.95 ^ab^
12	1.5	7.5	41	2.16 ± 0.20 ^a^	33 ± 3.64 ^abc^	71 ± 0.07 ^cd^	105 ± 3.7 ^ab^	122 ± 4.76 ^ab^
13	1.5	7.5	41	2.25 ± 0.12 ^a^	33 ± 2.32 ^abc^	71 ± 0.94 ^cd^	109 ± 8.5 ^ab^	119 ± 7.35 ^ab^

All values are means of three replications ± standard deviation. Values with the same letter(s) within a column are not significantly different. *^,^ **^,^ *** significantly differ at *p* > 0.05, *p* < 0.01, and *p* < 0.001, respectively.

**Table 4:** The experimental values of the physicochemical variables for each independent variable.

Experiment No.				*WAI*	*WSI* (%) ***	ΔΕ ***	*BI* **
	HPE (g)	GW (g)	*Sunsik* (g)				
1	1	5	44	1.88 ± 0.06	42 ± 1.92 ^d^	0.72 ± 0.18 ^ab^	19.4 ± 0.35 ^c^
2	2	5	43	1.95 ± 0.04	41 ± 1.01 ^d^	0.22 ± 0.06 ^bc^	20.2 ± 0.38 ^ab^
3	1	10	39	1.91 ± 0.02	41 ± 0.49 ^d^	1.12 ± 0.24 ^abc^	20.0 ± 0.39 ^abc^
4	2	10	38	1.82 ± 0.12	40 ± 0.33 ^d^	0.48 ± 0.08 ^b^	20.1 ± 0.20 ^ab^
5	0.79	7.5	41.71	1.86 ± 0.04	41 ± 1.17 ^d^	1.13 ± 0.27 ^ab^	19.5 ± 0.34 ^bc^
6	2.21	7.5	40.29	1.91 ± 0.02	40 ± 1.26 ^d^	0.39 ± 0.12 ^b^	20.4 ± 0.32 ^a^
7	1.5	3.96	44.54	1.92 ± 0.08	32 ± 0.23 ^e^	0.43 ± 0.04 ^b^	20.3 ± 0.31 ^ab^
8	1.5	11.04	37.46	1.89 ± 0.02	59 ± 0.63 ^a^	0.56 ± 0.16 ^b^	19.9 ± 0.23 ^abc^
9	1.5	7.5	41	1.86 ± 0.06	49 ± 1.12 ^c^	0.23 ± 0.02 ^bc^	20.2 ± 0.23 ^ab^
10	1.5	7.5	41	1.89 ± 0.05	53 ± 2.11 ^b^	0.06 ± 0.03 ^c^	20.2 ± 0.05 ^ab^
11	1.5	7.5	41	1.83 ± 0.05	46 ± 1.89 ^c^	0.24 ± 0.04 ^bc^	20.0 ± 0.11 ^abc^
12	1.5	7.5	41	1.83 ± 0.01	48 ± 1.95 ^c^	0.29 ± 0.07 ^bc^	19.8 ± 0.09 ^abc^
13	1.5	7.5	41	1.84 ± 0.08	46 ± 1.93 ^c^	0.30 ± 0.06 ^bc^	19.9 ± 0.01 ^abc^

All values are means of three replications ± standard deviation. Values with the same letter(s) within a column are not significantly different. **^,^ *** significantly differ at *p* < 0.01 and *p* < 0.001, respectively.

**Table 5:** Predicted and actual values of the optimized *Sunsik* formulation.

Responses	Optimized Formulation			
	Goal	Predicted Values	Actual Values	Error (%)
GABA (Y_1_, μg/g)	Maximize	2.21	2.23 ± 0.04	0.9
TFC (Y_2_, μg CE g)	Maximize	33.39	33.75 ± 0.25	1.07
TPC (Y_3_, μg GE/100g)	Maximize	72.56	73.26 ± 0.46	0.97
DPPH (Y_4_, µM TE/100g)	Maximize	110	112 ± 0.58	2.12
TEAC (Y_5_, mM TE/100g)	Maximize	124	125 ± 0.58	0.54
WAI (Y_6_)	Minimize	1.84	1.80 ± 0.03	2.44
WSI (Y_7_)	Maximize	49.2	48.52 ± 1.28	0.25
∆E (Y_8_)	Minimize	0.38	0.25 ± 0.03	11.71
BI (Y_9_)	Minimize	20.06	20.42 ± 0.12	1.61

**Table 6:** Health-conscious and physicochemical properties of the optimized Sunsik formulation.

		Commercial *Sunsik*	Optimized *Sunsik*
Health conscious properties	GABA (*μg/g*) ***	1.7 ± 0.09 ^b^	2.23 ± 0.04 ^a^
	TFC (*μg* *CE/g*) ***	19.8 ± 1.72 ^b^	33.75 ± 0.25 ^a^
	TPC (*μg* *GE/100g*) ***	54.4 ± 3.57 ^b^	73.26 ± 0.46 ^a^
	DPPH (*µM TE/100g*) ***	77.3 ± 2.06 ^b^	112 ± 0.58 ^a^
	TEAC (*mM TE/100g*) ***	96.9 ± 3.27 ^b^	125 ± 0.58 ^a^
	EC_50_ for Caco-2 cell (mg/mL) ***	97.4 ± 4.2 ^a^	45.7 ± 1.6 ^b^
	EC_50_ for HepG2 cell (mg/mL) ***	76.2 ± 3.8 ^a^	35.2 ± 2.5 ^b^
Physicochemical properties	WAI ***	3.6 ± 0.03 ^a^	1.80 ± 0.03 ^b^
	WSI (%) ***	7.4 ± 0.1 ^b^	48.52 ± 1.28 ^a^
	Apparent viscosity (cP) ***	294 ± 2.87 ^a^	47 ± 4.42 ^b^

All values are means of three replications ± standard deviation. Values with same letter(s) within a row are not significantly different. *** significantly differ at *p* < 0.001.
